# Effect of extended intravenous diclofenac infusions on brain tissue oxygenation in patients with acute brain injury

**DOI:** 10.1186/s40635-025-00759-3

**Published:** 2025-05-13

**Authors:** Julian Klug, David Cortier, Stefan Wolf, Emmanuel Carrera, Charles Cerf, Urs Pietsch

**Affiliations:** 1 Department of Intensive Care, Foch Hospital, Suresnes, France; 2 Stroke Research Group, Department of Clinical Neurosciences, University Hospital and Faculty of Medicine, Rue Gabrielle-Perret-Gentil 4, 1211 Geneva, Switzerland; 3 Department of Neurosurgery, Charité-Universitätsmedizin Berlin, Berlin, Germany; 4 Department of Emergency Medicine, Inselspital, Bern University Hospital, University of Bern, Bern, Switzerland; 5 Division of Perioperative Intensive Care Medicine, Cantonal Hospital St. Gallen, Rorschacher Strasse 95, 9007 St. Gallen, Switzerland

**Keywords:** Brain tissue oxygen, Diclofenac, Fever, Temperature control, Traumatic brain injury, Subarachnoid hemorrhage

## Abstract

**Background:**

Fever is associated with worse outcomes in patients with acute brain injury. Diclofenac, a non-steroidal anti-inflammatory drug, is commonly used as antipyretic therapy. As evidence emerged that short diclofenac infusions (< 1 h) decrease brain tissue oxygen (PtO2) and cerebral perfusion pressure (CPP), clinical practice has shifted to extended infusions (12 h). The purpose of this study was to investigate the effects of extended diclofenac infusion for the treatment of fever on cerebral perfusion and tissue oxygenation after acute brain injury.

**Results:**

We conducted a retrospective study of prospectively collected data from a cohort of 18 patients with acute brain injury and PtO2 monitoring admitted between November 2018 and April 2024. The hour before and the 12 h during an extended diclofenac infusion were compared. Additionally, we compared the 12 h prior and 12 h during the diclofenac infusion. Cerebral autoregulation and metabolites obtained by microdialysis were assessed in a subgroup of patients. Thirty-nine interventions were analyzed. Core temperature decreased from 38.1°C in the hour before to 37.4 °C during an extended diclofenac infusion (*p* < 0.0001). ICP (11.0 vs 10.0 mmHg, *p* < 0.0001) and heart rate (84 vs. 77 bpm, *p* < 0.0001) decreased. CPP and PaCO2 did not vary significantly. PtO2 decreased from 23.1 mmHg (IQR 19.0–31.4) during fever peak to 21.7 mmHg (IQR 17.8–27.2) (*p* < 0.0001). Median PtO2 during the 12 h before diclofenac was 23.3 mmHg (IQR 18.9–30.5). In a multivariable analysis the effect of treatment was significantly influenced by heart rate and temperature (*p* < 0.0001).

**Conclusions:**

Extended diclofenac infusions for the treatment of fever in patients with acute brain injury achieve a clinically significant reduction in temperature but are associated with a small decrease in PtO2, even in the setting of maintained CPP.

## Background

Fever occurs in over 50% of patients with acute brain injury admitted to the intensive care unit (ICU) [1]. Even small temperature elevations are correlated with worse prognosis [2–4]. Fever correlates with cerebral oxygen consumption, disrupts the blood–brain barrier, and increases excitatory neurotransmitters and free radicals [5]. This results in decreased cerebral blood flow, cerebral edema, and elevated intracranial pressure [6, 7].

Current consensus guidelines recommend controlled normothermia (36.0–37.5 °C) after acute neurovascular and traumatic brain injury [8, 9]. Targeted management relies on a tiered multimodal strategy [10]. Paracetamol is the first-line pharmacologic option, but often insufficient for temperature control alone [11]. Cooling devices are frequently used in this setting but are associated with patient discomfort and shivering, which can lead to discontinuation of therapy or the initiation of sedation or even muscle relaxants [10, 12]. Further pharmacologic options include metamizole, which is not available in many countries, and non-steroidal anti-inflammatory drugs [13].

Diclofenac, a selective COX-2 inhibitor, has been shown to be highly effective in reducing temperature [14]. Its use as a short infusion (≤ 1 h) for the treatment of pyrexia has been however associated with a decrease in cerebral perfusion pressure (CPP) and was associated with reduced brain tissue oxygen (PtO2) [11, 15]. This data has prompted clinicians to abandon its use or to favor extended (≥ 12 h) over short infusions [16–18]. However, the effect of this administration protocol on PtO2 has not yet been investigated.

In this study, we aimed to describe the effects of extended intravenous diclofenac infusions on cerebral tissue oxygenation and cerebral perfusion pressure in patients with acute brain injury admitted to the ICU.

## Methods

### Study setting and design

This study was designed as a retrospective cohort study based on prospectively collected data for the evaluation of the effect of extended diclofenac infusions on cerebral tissue oxygenation. The study was performed at the ICU of the Cantonal Hospital St. Gallen, Switzerland. The study was conducted in accordance with the Helsinki declaration and approved by the local institutional review board (EKOS22/179 and EKOS 22/198). Consent was waived in accordance with Article 34 of the Swiss Federal Act on Human Research. This manuscript adheres to the Strengthening the Reporting of Observational Studies in Epidemiology (STROBE) guidelines [19].

### Patients

We enrolled 18 consecutive patients with acute neurologic injury receiving extended diclofenac infusions and concomitant monitoring of cerebral tissue oxygenation between November 2018 and April 2024. Events in which diclofenac was infused in less than 1 h, or with diclofenac use in the preceding 12 h were excluded. Patients were managed according to current guidelines [20–22]. Normothermia (36.0–37.5 °C) was targeted with a stepwise protocol. All patients received paracetamol and metamizole as first-line treatment. Extended diclofenac infusions were used as second-line therapy, with a protocolized 75 mg infused over 12 h. This duration was chosen as a balance between previously described continuous infusions [16–18] and practicality, as well as to match the cyclical nature of hyperthermia. Blood pressure was measured invasively using arterial catheters zeroed at the level of the foramen of Monro to assess CPP.

Cerebral tissue oxygenation was measured using intraparenchymal probes (Licox, Integra LifeSciences, Ratingen, Germany or NEUROVENT-PTO, Raumedic, Helmbrechts, Germany) placed in locations at greatest risk for secondary brain injury. Location was confirmed by head CT-scan. Intracranial pressure (ICP) was measured using intraparenchymal catheters. Body temperature was measured using a bladder catheter equipped with temperature sensors. Arterial carbon dioxide content (PaCO2) was assessed using regular blood gas analysis. In a subset of patients, extracellular metabolic components (glucose, lactate, pyruvate) were analyzed using cerebral microdialysis (ISCUSflex Microdialysis Analyzer, M Dialysis AB, Sweden) to derive the lactate–pyruvate ratio (LPR), a marker of cellular redox status [23], and/or cerebro-vascular autoregulatory status was monitored using the pressure reactivity index (PRx) [24] (CNS Envision, Moberg Analytics, Philadelphia, USA). Multimodal cerebral monitoring data were recorded using a bedside integrative platform (Moberg Analytics, Philadelphia, USA). Patient records were retrospectively screened for other factors of influence during the period of drug administration, including vasospasm related delayed cerebral ischemia, induced hyperoxia and changes in target temperature during external cooling. Data with any coinciding external factor were excluded.

### Adverse events

Patient records were retrospectively screened for the occurrence of bleeding, acute kidney injury, major adverse cardiovascular events as well as hypersensitivity reactions in the 48 h after every diclofenac infusion. Acute kidney injury was defined as an increase of creatinine by 50% or 26 μmol/l from the baseline of the prior days or a decrease in output to < 0.5 ml/kg/h for 6 consecutive hours. Major adverse cardiovascular events were defined as the occurrence of myocardial infarction or acute decompensated heart failure. Hypersensitivity reactions were defined as the occurrence of exanthema, respiratory failure or cardiovascular collapse linked to the infusion.

### Statistical methods

We aimed to evaluate the effect of an extended diclofenac infusion on cerebral oxygenation and hemodynamics. We compared the hour before the onset of the infusion to the 12 h during the infusion, to capture the effect of diclofenac on the fever peak. We further compared the 12 h before the initiation of the drug to the 12 h after initiation, to compare the patients’ steady state before and after the start of the infusion. For univariable analysis, we used a mixed-effects model with, respectively, PtO2, CPP, temperature, heartrate, PaCO2, end-tidal CO2 (EtCO2), LPR and PRx as response variable, pre/post initiation of treatment status as fixed effects and subject as random effect. To control for the confounding effect of recorded hemodynamic variables on PtO2 we added a multivariable analysis. A mixed-effects model with PtO2 as dependent variable, CPP, temperature, heartrate, pre/post initiation of treatment status as well as their interactions as fixed effects and subject as random effect. Interactions were included to assess whether the relationship between treatment status and PtO2 is modified by a third variable. For multivariable analysis, variables with similar sampling rates were grouped as medians into time bins of 36 s. Variables not recorded for all patients were excluded from multivariable analysis and from the comparison with the fever peak. We performed a subgroup analysis in patients with traumatic brain injury (TBI) and patients with neurovascular injuries. Medians are used to represent central tendency and interquartile ranges (IQR) are used to represent variation across data. Statistical significance was assumed at a p level of < 0.05. Imputing of missing data was not performed. We used Python (version 3.11.9) and statsmodels (version 0.14.2) for statistical analysis [25]. To obtain nonparametric confidence intervals for mean values used in the graphical representations, bootstrapping with replacement was used (1000 iterations).

## Results

Between November 18th 2018 and April 15th 2024, we screened 99 patients monitored with PtO2 probes, of whom 18 (18%) were included with a total of 39 administrations of an extended diclofenac infusion. Data from a one administration had to be excluded because of an extended period of systemic hyperoxia. Patients received a median of 1 (IQR 1–2) diclofenac infusions. The median age was 46 (IQR 34–51) and 5 (28%) patients were female. 10 (56%) patients were admitted for traumatic brain injury with a median injury severity scale of 29 (18–38). 8 (44%) were admitted for a neurovascular event of which 7 (88%) were subarachnoid hemorrhages. Median Glasgow coma scale at admission was 6 (IQR 3–9). 8 (44%) patients underwent a surgical and 7 (39%) a radiological intervention during their ICU stay. Median time from admission to first administration of diclofenac was 6.1 days (IQR 4.5–8.1) and median duration of diclofenac infusion was 12.03h (IQR 12.00–12.03). ICU and hospital length of stay were, respectively, 18 days (IQR 12–20) and 25 days (IQR 22–30). 3 (17%) patients died during the hospitalization. Baseline characteristics are reported in Table 1.

**Table 1:** Study population characteristics

Demographics	
Number of patients	18
Number of drug administrations	39
Age	46 (34–51)
Gender (female)	5 (28%)
Admission status	
Neurovascular	8 (44%)
TBI	10 (56%)
Initial GCS	6 (3–9)
Interventions	
Surgical	8 (44%)
Radiological	7 (39%)
Outcomes	
ICU length of stay (days)	18 (12–20)
Hospital length of stay (days)	25 (22–30)
In-hospital mortality	3 (17%)

Numbers are reported as median (interquartile ratio, IQR) and number (percentage, %)

*TBI* traumatic brain injury, *GCS* Glasgow coma scale, *ICU* intensive care unit

### Comparison of 1 h before with 12 h during the extended diclofenac infusion

**Fig. 1:**
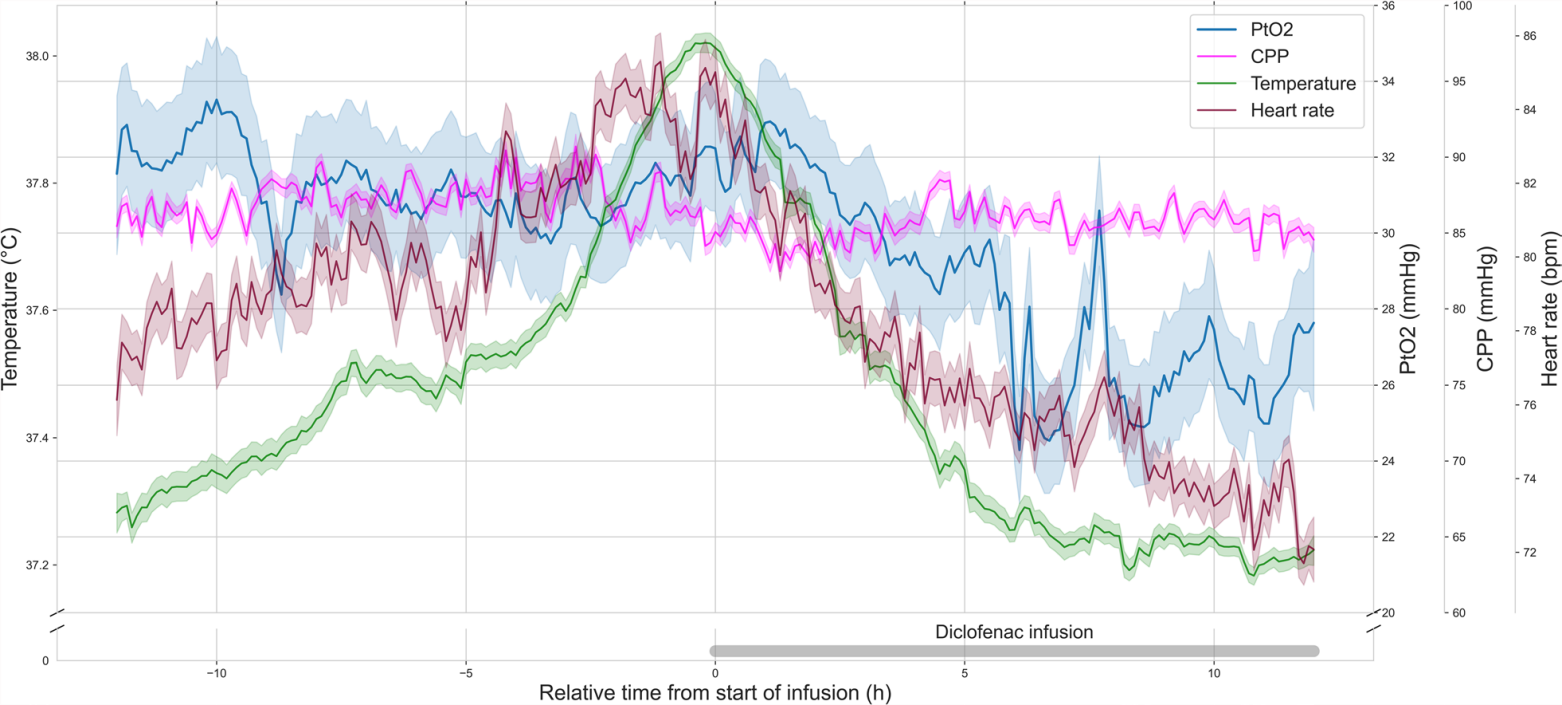
Systemic and intra-cerebral hemodynamic indices before and after the initiation of an extended diclofenac infusion. Temperature (green), PtO2 (blue), CPP (magenta) and heart rate (red) during the 12 h before and after the start of an extended diclofenac infusion (75 mg over 12 h, grey bar). The relative time to the start of the diclofenac infusion is represented on the x-axis (hours). Temperature (°C), PtO_2_ (mmHg), CPP (mmHg) and heart rate (bpm) are represented on the 4 separate y-axes. Lines represent the mean value in time bins of 6 min (*t* = 241) for all patients (*n* = 18). Shaded areas represent the 95% confidence intervals of the mean obtained through bootstrapping (*n* = 1000). After the start of the infusion, temperature, heart rate and PtO2 decrease concomitantly. *CPP* decreases minimally. *PtO2:* cerebral tissue oxygen pressure, *CPP:* cerebral perfusion pressure, *bpm*: beats per minute

Core temperatures significantly decreased from 38.1 °C (IQR 37.7–38.3) 1 h before infusion onset to 37.4 °C (IQR 37.1–37.7) during the extended diclofenac infusion (*p* < 0.0001). Concomitantly heart rate (84.0 vs. 77.0 bpm, *p* < 0.0001) as well as ICP (11.0 vs 10.0 mmHg, *p* < 0.0001) decreased significantly. CPP (84 vs. 85 mmHg, *p* = 0.58), PaCO2 (36.1 vs. 36.4 mmHg, *p* = 0.74) and EtCO2 (36 vs. 34 mmHg, *p* = 0.22) did not vary significantly. During the intervention, PtO2 significantly decreased from 23.1 mmHg (IQR 19.0–31.4) to 21.7 mmHg (IQR 17.8–27.2) in both univariable and multivariable analysis (*p* < 0.0001) (Fig. 1). In multivariable analysis, the effect of the diclofenac infusion was significantly modified by heart rate, CPP and temperature (*p* < 0.0001).

### Comparison of 12 h before with 12 h during the extended diclofenac infusion

**Fig. 2:**
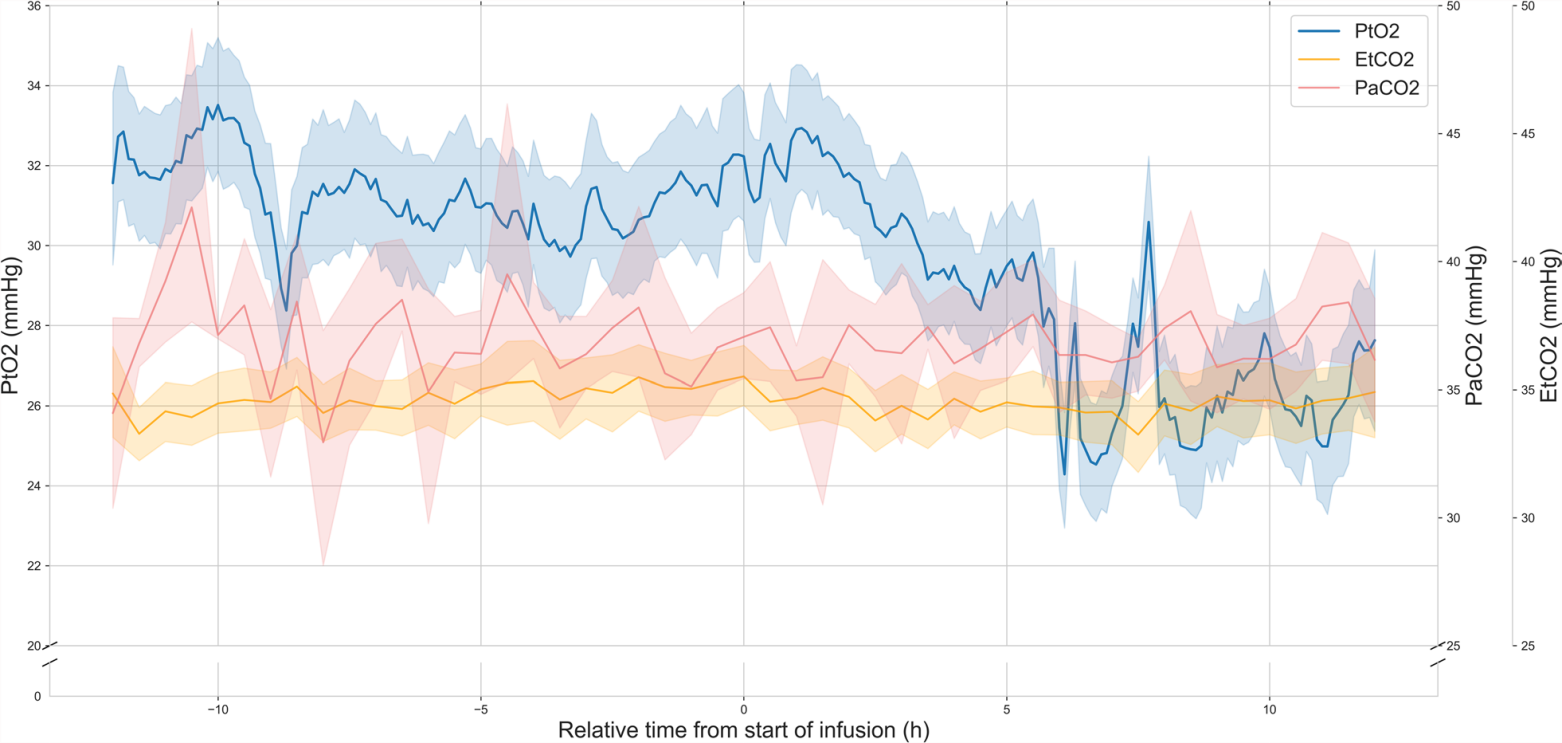
Brain tissue oxygen tension, arterial and end-tidal CO_2_ before and after the initiation of an extended diclofenac infusion. PtO2 (blue), PaCO2 (red) and EtCO_2_ (orange) during the 12 h before and after the start of an extended diclofenac infusion (75 mg over 12 h). The relative time to the start of the diclofenac infusion is represented on the x-axis (hours). PtO_2_ (mmHg), PaCO_2_ (mmHg) and EtCO_2_ (mmHg) are represented on the 3 separate y-axes. Lines represent the mean value in time bins of 6 min (PtO_2_) (t = 241) or 30 min (PaCO_2_ & EtCO_2_) (*t *= 49) for all patients (*n* = 18). Shaded areas represent the 95% confidence intervals of the mean obtained through bootstrapping (*n* = 1000). After the start of the infusion, PaCO2 remains constant (*p* = 0.5703), whereas EtCO_2_ slightly decreases (*p* < 0.0001). *PtO*_*2*_: cerebral tissue oxygen pressure, *PaCO2:* partial arterial pressure of carbon dioxide, *EtCO*_*2*_: end-tidal partial pressure of carbon dioxide

**Fig. 3:**
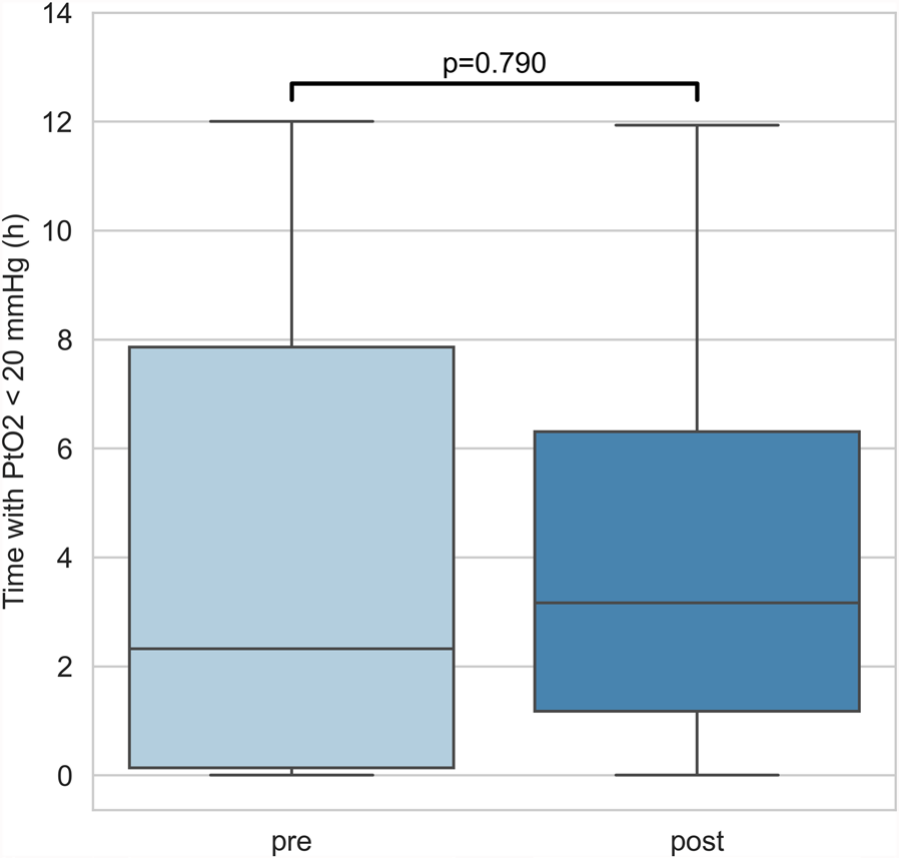
Time spent under an ischemic threshold of PtO2 before and after the initiation of an extended diclofenac infusion. Boxplot representing time spent under a threshold of PtO2 < 20 mmHg in hours during the 12 h before (light blue) and after (dark blue) the initiation of an extended diclofenac infusion for all patients (*n* = 18). The box spans from the lower to the upper quartile and a horizontal line represents the median. Whiskers indicate full range of data. The difference of time spent under an ischemic threshold is not statistically different before and after the onset of a diclofenac infusion (*p* = 0.7903)

When comparing variables during the infusion with the 12 h before infusion onset to capture a prior steady state, decrease in core temperatures (37.6 vs. 37.4 °C, *p* < 0.0001), in heart rate (81 vs. 77 bpm, *p* < 0.0001) and in ICP (11.0 vs 10.0 mmHg, *p* < 0.0001) remained significant. Change in CPP was statistically significant, but was numerically minimal with a median of 85.0 mmHg (IQR 76.0–97.5) before and 85.0 mmHg (IQR 75.0–96.0) after the initiation of the diclofenac infusion (*p* < 0.0001). PaCO2 remained constant at 36 mmHg (p = 0.5703) (Fig. 2). EtCO2 decreased from 35 mmHg (IQR 31–38) to 34 mmHg (IQR 31–37) (*p* < 0.0001). In the single variable analysis, PtO2 decreased significantly from 23.3 mmHg (IQR 18.9–30.5) to 21.7 mmHg (IQR 17.8–27.2) with the diclofenac infusion (*p* = 0.0003). This direct association was however not significant in the multivariable analysis (*p* = 0.1003). When considering the effects of the diclofenac infusion, heart rate, CPP and temperature, only the interaction of treatment and heart rate (*p* = 0.0412), as well as the interaction between treatment, temperature and heart rate (*p* = 0.0351) had a significant effect on PtO2 (Fig. 1). In other words, the effect of treatment on PtO2 was not significant on its own when considering confounding variables but was significantly modified by heart rate and temperature. The decrease of PtO2 was present in both the TBI (19.5 vs. 19.1 mmHg, *p* < 0.0001) and neurovascular injury subgroups (29.3 vs. 25.9 mmHg, *p* < 0.0001) in multivariable analysis. Time spent under a threshold of 20 mmHg did not vary significantly (2.32 vs. 3.17 h, *p* = 0.7903) (Fig. 3).

**Fig. 4:**
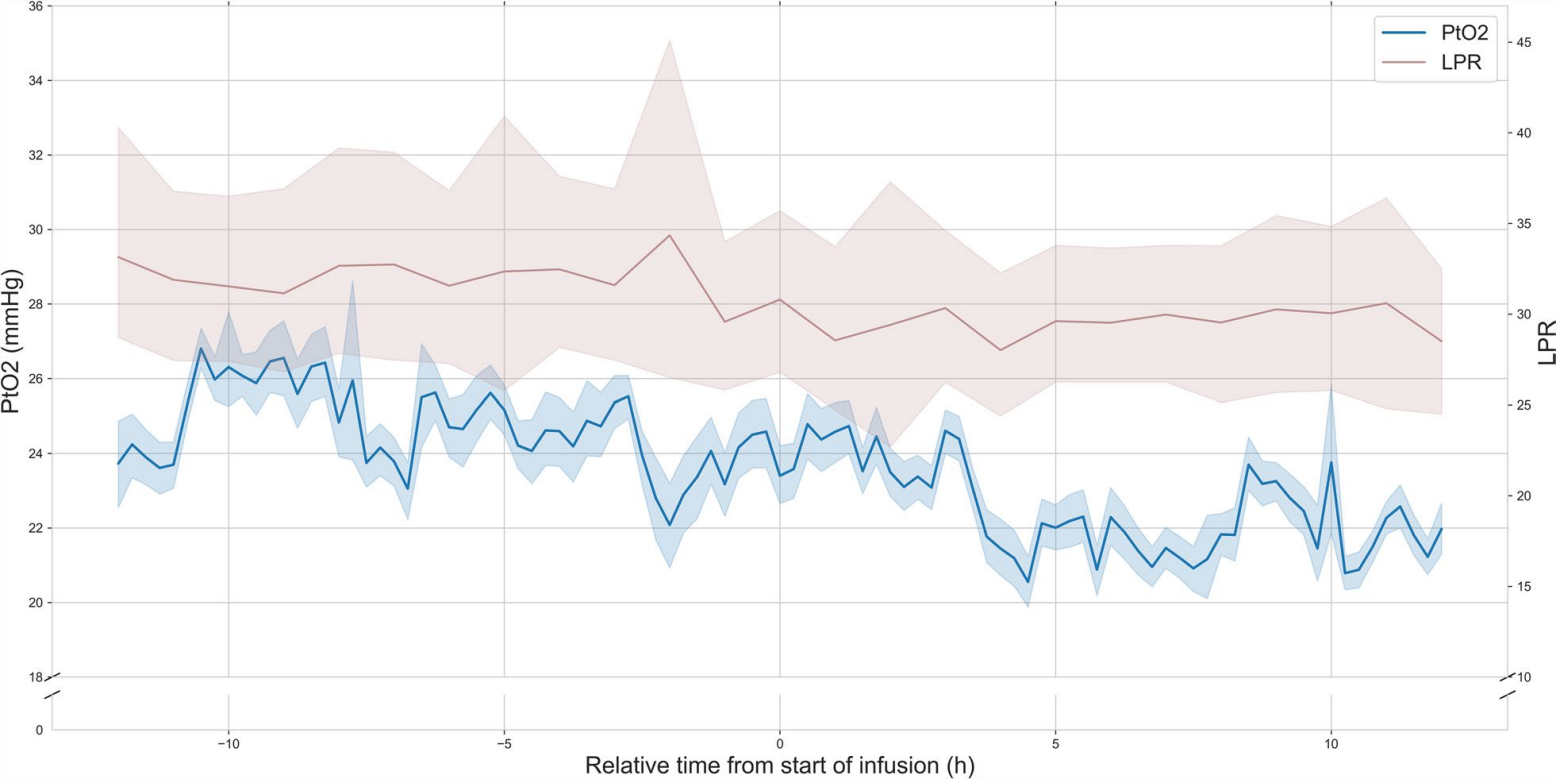
Brain tissue oxygen tension and lactate–pyruvate ratio before and after the initiation of an extended diclofenac infusion. PtO2 (blue) and LPR (dark red) in a subset of patients (*n* = 8) with cerebral microdialysis monitoring during the 12 h before and after the start of an extended diclofenac infusion (75 mg over 12 h). The relative time to the start of the diclofenac infusion is represented on the x-axis (hours). PtO2 (mmHg), and LPR are represented on the 2 separate y-axes. Lines represent the mean value in time bins of 6 min (*t *= 241). Shaded areas represent the 95% confidence intervals of the mean obtained through bootstrapping (*n* = 1000). After the start of the infusion, both PtO2 and LPR decrease (*p* = 0.0003). Although LPR is globally elevated, the decrease In PtO2 is not accompanied by metabolic ischemia. *PtO2:* cerebral tissue oxygen pressure, *LPR:* lactate–pyruvate ratio

**Fig. 5:**
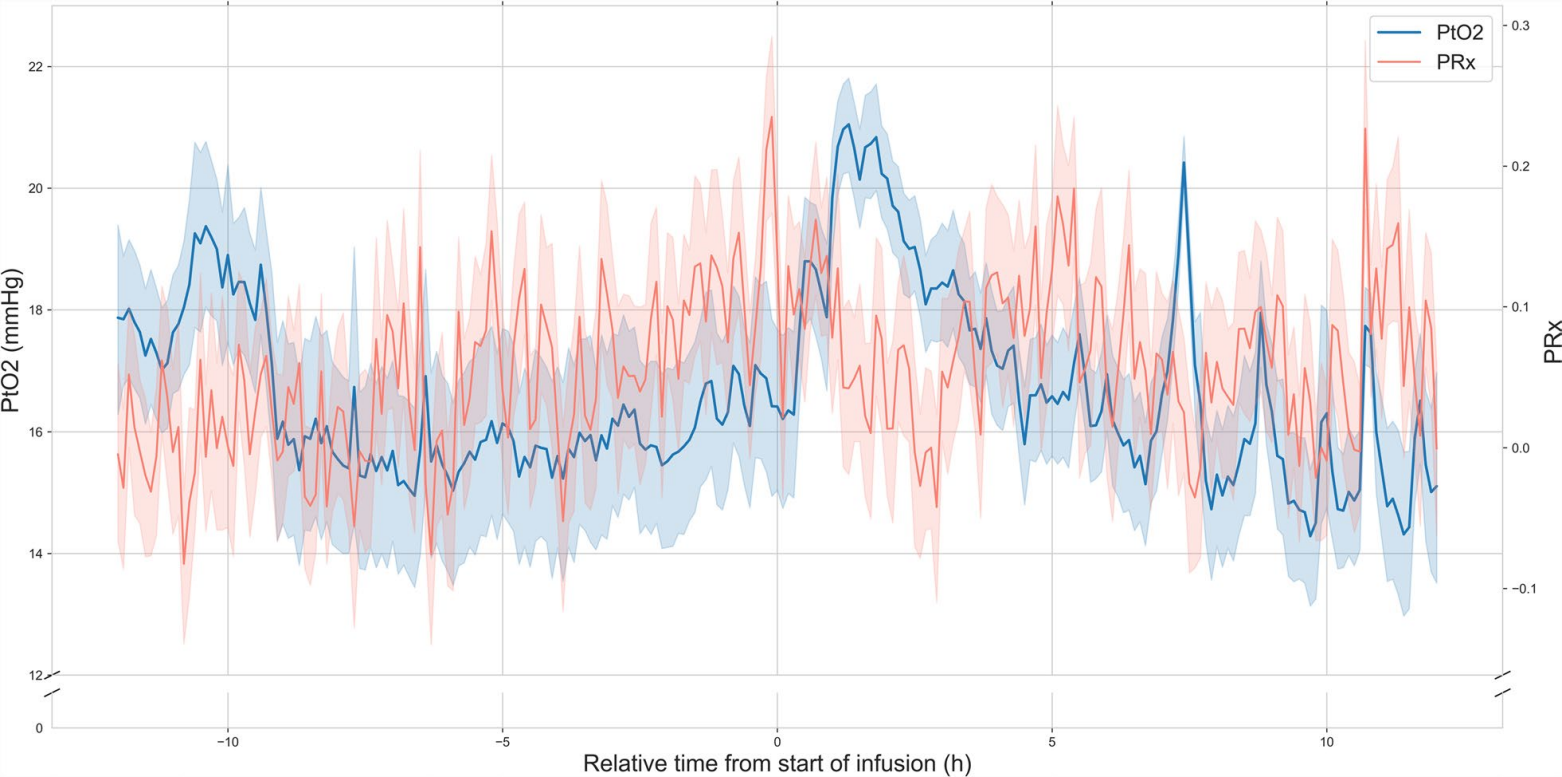
Brain tissue oxygen tension and pressure reactivity index before and after the initiation of an extended diclofenac infusion. PtO2 (blue) and PRx (red) in a subset of patients (*n* = 15) with autoregulation monitoring during the 12 h before and after the start of an extended diclofenac infusion (75 mg over 12 h). The relative time to the start of the diclofenac infusion is represented on the x-axis (hours). PtO2 (mmHg), and PRx are represented on the 2 separate y-axes. Lines represent the mean value in time bins of 6 min (*t *= 241). Shaded areas represent the 95% confidence intervals of the mean obtained through bootstrapping (*n* = 1000). After the start of the infusion, PRx increases minimally (*p* = 0.0186), within the normal range. *PtO2* cerebral tissue oxygen pressure, *PRx:* pressure reactivity index

LPR and PRx were recorded in 8 and 15 of the 18 patients, with, respectively, 14 and 30 drug administrations. After the onset of the diclofenac infusion, LPR decreased from 31 (IQR 25–35) during the 12 h prior to 29 (IQR 24–33) (*p* = 0.0003) (Fig. 4). PRx increased from 0.03 (IQR − 0.23–0.32) to 0.06 (IQR − 0.22–0.34) (*p* = 0.0186) (Fig. 5).

In two patients, acute kidney injury was recorded in the 48 h following the infusion. In both cases, recovery was spontaneous, and no dialysis was required. No bleeding, major cardiovascular events or hypersensitivity reactions were recorded (Table 2).

**Table 2:** Adverse events

Event	*n* (%)
Bleeding	0 (0)
Acute kidney in	2 (11)
Major cardiovascular event	0 (0)
Hypersensitivity reaction	0 (0)

Adverse events identified in the 48 h after a diclofenac infusion

## Discussion

In this single-center retrospective cohort study of patients admitted to the ICU after acute brain injury an extended diclofenac infusion for the treatment of fever achieved a clinically significant reduction in temperature but was associated with a small decrease in PtO2.

We observed a mean decrease in PtO2 of 9.6%, smaller than the 13% reported reduction following short diclofenac infusions in previous studies [11, 15]. The clinical relevance of this effect on PtO2 is however uncertain. In an exploratory subgroup analysis, we show that the decrease of PtO2 is associated with a small decrease in LPR, and thus no increase of metabolic markers of ischemia. This could signal that although oxygen delivery might be reduced, oxygen demand is equally scaled down or extraction proportionally more efficient with the decrease in temperature. This hypothesis is supported by previous studies reporting a decrease in PtO2 after administration of paracetamol, metamizole [11], and with the application of automated surface cooling [26]. The effect of spontaneous defervescence is unknown, although a spontaneous increase in temperature has been associated with an increase in PtO2 [27]. Interestingly, observational data even linked the use of non-steroidal anti-inflammatory drugs with improved outcomes after aneurysmal subarachnoid hemorrhage [28].

We observed no clinically significant change in ICP and no reduction in CPP after extended diclofenac infusion contrasting with the reported effect of short infusions (mean difference 0.58% vs. 6.3–10%) [11, 15]. There was no association of post-treatment CPP with the reduction in PtO2, confirming prior findings [11]. Perfusion pressure is thus unlikely to explain the effect on cerebral tissue oxygenation. Although we observed a statistically significant increase in PRx in a subset of patients, it was numerically small and unlikely to explain the decrease in PtO2. In prior studies, the change in PtO2 was not significantly associated with impaired autoregulation status [11]. In our study, PaCO2 did not change significantly, rendering a CO2-mediated vasoactive effect unlikely. In a multivariable analysis, we found only the interactions of treatment, heart rate and temperature to be significantly associated with PtO2. A reduction in heart rate could lead to a reduction in cardiac output, cerebral blood flow and thus cerebral oxygen supply. As metabolic markers of ischemia do not increase, a resolution of fever mediated tachycardia may induce a reduction of inappropriately high cardiac output and by consequence a normalization of cerebral perfusion. No patient underwent concomitant cardiac output monitoring. Although a small reduction in EtCO2 was observed, this is unlikely to be associated with a low cardiac output. Alternatively, the decrease in PtO2 could be due to neurovascular coupling, where the brain adjusts cerebral blood flow to match its energy needs. Lowering body temperature may reduce cerebral energy demand, leading to a decrease in CBF [7, 29]. Intra-cellular alterations, as well as a reduction in sympathetic tone or inflammatory mediators, which can influence vascular resistance, may contribute to the effect [30]. Concurrently, in our study, we observed a PRx in the normal range suggesting that autoregulation is preserved. The temperature-dependent change in hemoglobin–oxygen dissociation may also influence PtO2 [31]. Regional distribution of blood flow was shown to be altered by temperature in prior functional imaging studies [32]. As the assessment of brain tissue oxygenation is restricted to a small volume of brain tissue surrounding the tip of the probe, redistribution of blood flow could alter PtO2 measures and uncouple it from global cerebral blood flow.

Adverse events associated with extended diclofenac infusions were rare in this study. Non-steroidal anti-inflammatory drugs are well known risk factors of acute kidney injury with a reported odds ratio of 1.2 to 1.7 [33–35] and the association of the two events of transient worsening of kidney function with the diclofenac infusion cannot be excluded. The risk of cardiovascular and bleeding events is dose-dependent and rare with daily doses under 150 mg [36]; no events were recorded in this study.

This work has several limitations and therefore all findings should be interpreted with caution. Firstly, our study is retrospective, mono-centric and only included a small number of patients. Consequently, our data do not allow to infer definitive causal relationships and there is little data to support our mechanistic hypotheses. Not all patients had monitoring of cerebral autoregulation and metabolism, and cardiac output monitoring was not performed. Many external and pathophysiological phenomena can alter PtO2, and not all confounding factors can be excluded in this retrospective study. Long-term outcomes were not assessed, and an observational study is insufficient to compare the effects of different temperature control strategies on outcome, which can only be demonstrated by randomized controlled trials. The optimal duration of an extended diclofenac infusion is unknown, and while previous studies have used continuous infusions over 24 h to several days [16–18], only a 12-h protocol was evaluated in this work.

Finally, the impact of fever management on outcome after acute brain injury is still unclear. Although observational data suggest an association of fever with worse outcomes, the recently published randomized INTREPID trial decreased fever burden by use of a mechanical temperature management system, but failed to show a benefit of fever prevention on functional outcome at 3 months [12]. Indeed, fever episodes may not always be associated with impaired cerebral metabolism and might reflect a more complex interplay of infection, neuroinflammation, cerebral perfusion, and metabolic demands, underscoring the need for further research to clarify when and how temperature management might offer a meaningful benefit. This exploratory study adds to the evidence that extended diclofenac infusions are safe and result in a measurable but probably clinically insignificant decrease in PtO2. Accordingly, extended diclofenac infusions may be considered as part of a multimodal approach to optimize fever control in patients with acute brain injury that could be evaluated in a future clinical trial.

## Conclusions

In this exploratory study, we report that extended diclofenac infusions effectively control fever and are associated with a modest reduction in PtO2. This decrease is less pronounced than previously reported with short infusion protocols. These findings call into question the clinical relevance of the observed PtO2 variation. In this setting, clinicians must balance the risks and benefits of a small decrease PtO2 through drug-based therapy, with the complications of device cooling or the effect of higher temperatures.

## Data Availability

The computer code used in this study is open-source and available at https://github.com/JulianKlug/neuropyro. The datasets generated and analyzed during the current study are not publicly available to protect patient confidentiality but are available from the corresponding author on reasonable request.

## Abbreviations

ICU: Intensive care unit
PtO2: Partial pressure of brain tissue oxygen
CPP: Cerebral perfusion pressure
LPR: Lactate–pyruvate ratio
PRx: Pressure reactivity index
PaCO2: Arterial partial pressure of carbon dioxide
EtCO2: End-tidal carbon dioxide
IQR: Interquartile range
bpm: Beats per minute
